# Prognostic value of functional SMAD4 localization in extrahepatic bile duct cancer

**DOI:** 10.1186/s12957-022-02747-3

**Published:** 2022-09-10

**Authors:** Hirotoshi Takayama, Shogo Kobayashi, Kunihito Gotoh, Kazuki Sasaki, Yoshifumi Iwagami, Daisaku Yamada, Yoshito Tomimaru, Hirofumi Akita, Tadafumi Asaoka, Takehiro Noda, Hiroshi Wada, Hidenori Takahashi, Masahiro Tanemura, Yuichiro Doki, Hidetoshi Eguchi

**Affiliations:** 1 Department of Gastroenterological Surgery, Graduate School of Medicine, Osaka University, Suita, Japan; 2 Department of Surgery, National Hospital Organization Osaka National Hospital, Osaka, Japan; 3 Department of Gastroenterological Surgery, Osaka International Cancer Institute, Osaka, Japan; 4 Department of Surgery, Osaka Police Hospital, Osaka, Japan; 5 Department of Surgery, Rinku General Medical Center, Osaka, Japan

**Keywords:** Extrahepatic biliary bile duct cancer, SMAD4, Intra-tumoral localization, Chemoradiation therapy

## Abstract

**Background:**

SMAD4 is a key mediator of TGFβ signaling and one of the mutated genes in extrahepatic bile duct cancer (eBDC). It has been also reported that SMAD4 has dual functions, in carcinogenesis via silencing and in tumor invasion/metastasis via signaling, depending on tumor stage. We previously visualized more nuclear transitioning functional SMAD4 at the tumor invasion front than the central lesion. So, we investigated the localization of functional SMAD4 (e.g., invasion area or metastasis lesion) and its association with chemotherapy and chemo-radiation therapy.

**Methods:**

We performed SMAD4 immunostaining on 98 resected eBDC specimens and evaluated the presence of the functional form of nuclear SMAD4 at the central lesion, invasion front, and metastatic lymph node. We also examined the influence on chemotherapy after recurrence (*n* = 33) and neoadjuvant chemo-radiation therapy (NAC-RT, *n* = 21) and the prognostic value of using retrospective data.

**Results:**

In 73 patients without NAC-RT, 8.2% had loss of SMAD4 expression and 23.3% had heterogeneous expression. Patients without SMAD4 expression at any site had significantly poorer overall survival (OS) than other patients (*P* = 0.014). Expression of SMAD4 at the invasion front was related to better survival (recurrence-free survival [RFS] *P* = 0.033; OS *P* = 0.047), and no SMAD4 expression at the metastatic lymph node was related to poorer OS (*P* = 0.011). The patients who had high SMAD4 expression had poorer prognosis after recurrence (RFS *P* = 0.011; OS *P* = 0.056). At the residual cancer in the resected specimen, SMAD4 was highly expressed after NAC-RT (*P* = 0.039).

**Conclusions:**

Loss of SMAD4 protein expression was a poor prognostic factor in eBDC at resectable stage. However, the intensity of functional SMAD4 in eBDC is a marker of resistance to chemo-radiotherapy and malignant potential at advanced stages.

**Supplementary Information:**

The online version contains supplementary material available at 10.1186/s12957-022-02747-3.

## Introduction

Biliary tract cancers (BTCs), which include intrahepatic bile duct cancer (BDC), extrahepatic BDC (eBDC), and gallbladder cancer, arise from the epithelium of the bile duct and a highly malignant neoplasm. Although curative resection is the only effective treatment, more than half of BTC patients cannot undergo surgery because they are diagnosed at an advanced stage [1]. In addition, there is a high relapse rate in patients who undergo curative resection [2, 3]. Gemcitabine plus cisplatin (GC) has been the standard chemotherapy treatment for advanced/recurrent BTCs based on the results from the ABC-02 trial and the BT-22 trial [4, 5]. Gemcitabine and S-1 combination therapy (GS) has been shown to not be inferior to GC therapy [6]. However, the recommended treatment options for unresectable or metastatic disease are limited, and the prognosis of these patients is poor, with a median overall survival (OS) of approximately 1 year [7].

BTC is a genetically diverse collection of cancers. Genomic and transcriptomic analysis of BTCs has been performed to understand the molecular landscape and to develop a new molecular targeted therapy [8–11]. *KRAS*,* TP53*,* ARID1A*, and *SMAD4* have been identified as the most prevalent mutations in eBTC [12]. SMAD4 is a key mediator of the TGFβ signaling pathway [13] and works via nuclear transition. The protein functions as a tumor suppressor and inhibits cell proliferation. Mutations and deletions of *SMAD4* have been most commonly documented in pancreatic adenocarcinoma [14] and biliary tract cancer and colorectal cancer [15]. Furthermore, the loss of SMAD4 protein expression has been shown to correlate with poor prognosis in pancreatic, appendiceal, and esophageal adenocarcinomas. We previously showed that SMAD4 contributes to chemoresistance in BTCs by inducing epithelial-mesenchymal transformation (EMT) [16].

The TGFβ signaling pathway plays a dual role as both a tumor-suppressor and tumor-promotor depending on the tumor stage and tumor microenvironment [17]. Genomic alteration of genes encoding components of the TGFβ pathway, including *SMAD4*, has been observed frequently in hepatobiliary cancer. We previously visualized more nuclear transitioning functional SMAD4 at the tumor invasion front than the central lesion [16]. We thought that this intra-tumoral heterogeneity of functional SMAD4 was induced by tumor progression and the effect of SMAD4 on tumor progression depends on tumor stage. However, the significance of functional SMAD4 localization has not been examined in any detail.

Thus, our objective in the present study was to investigate the localization of functional SMAD4 in BTC and its significance using resected specimens. We also examined the association between functional SMAD4 and chemotherapy and radiotherapy.

## Materials and methods

### Resected specimens and patient characteristics

**Fig. 1:**
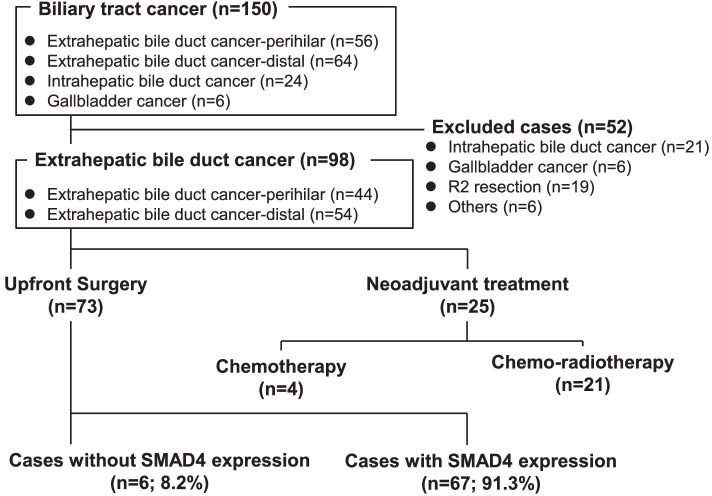
Flowchart of patient inclusion in this study

We retrospectively analyzed 98 cases of eBDC including 54 perihilar bile duct cancer and 44 distal bile duct cancer who underwent R0 or R1 resection between 2004 and 2018 at Osaka University Hospital or Osaka International Cancer Institute in Osaka, Japan. The race/ethnicity of all patients in this cohort is Japanese/Asian. Patients who underwent R2 resection were excluded from this study (Fig. 1). Resected specimens were formalin-fixed and preserved in paraffin blocks prior to immunohistochemistry. The use of resected samples was approved by the Human Ethics Review Committee of the Graduate School of Medicine, Osaka University (No. 20493). Written informed consent was obtained from all patients included in the study.

### Pre-operative treatment and follow-up treatment after surgery

After routine examination of the general condition, we performed computed tomography (CT) or magnetic resonance imaging, endoscopic retrograde cholangiography, and/or percutaneous transhepatic cholangiography, electrogram, spirogram, and chest X-rays. Preoperative staging was performed using image-based diagnosis. The treatment procedure was determined by the cancer board at each institution, consisting of radiologists, gastroenterologists, hepatologists, oncologists, and surgeons. After surgery, patients regularly underwent CT and the measurement of serum carcinoembryonic antigen (CEA) or carbohydrate antigen 19–9 (CA19-9) every 3 months in the first 2 years and every 6 months thereafter. If recurrence was clinically suspected, additional blood tests and imaging were performed to confirm recurrence. Recurrence was diagnosed based on these findings. After recurrence, we treated patients with chemotherapy, radiation, surgery, or best supportive care depending on the patients’ condition and the site and number of recurrences.

### Immunohistochemistry

Immunohistochemical staining for SMAD4 was carried out as described previously. In summary, resected specimens were cut into 3.5-µm slices, deparaffinized with xylene and ethanol, and bathed in citrate buffer at 110 °C for 20 min for antigen retrieval. Endogenous peroxidase activity was inhibited by treating the tissue sample with 3.0% hydrogen peroxidase solution in methanol for 20 min. Non-specific binding sites were blocked in 1 mol/L PBS with 10% normal goat serum from the Avidin/Biotin Blocking Kit (Vector Laboratories Inc., Burlingame, CA, USA). The slices were incubated at 4 °C overnight with anti-SMAD4 antibody (mouse monoclonal antibody, 1:50 dilution, Santa Cruz Biotechnology, USA). After washing with PBS, sections were loaded with secondary antibody from the Avidin/Biotin Blocking Kit (Vector Laboratories) for 1 h. Sections were stained with avidin–biotin complex reagents (Vector Laboratories) and 3,3′-diaminobenzidine (DAB) and counter-stained with hematoxylin. Finally, sections were dehydrated in graded concentrations of ethanol and xylene and mounted.

### Evaluation of immunohistochemistry

**Fig. 2:**
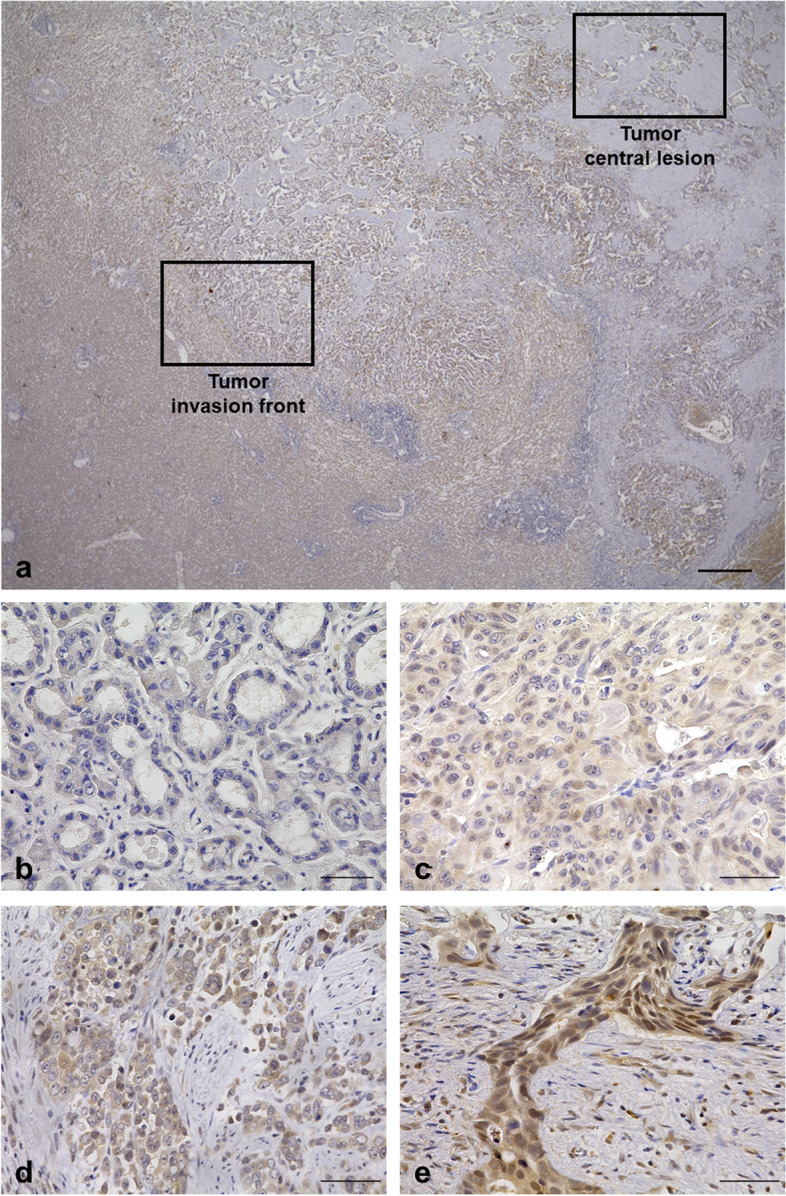
The definition of tumor invasion front and tumor central lesion, and typical immunohistochemical labeling for SMAD4 in extrahepatic biliary tract cancer. **a** The definition of tumor invasion front and tumor central lesion. Scale bar = 500 µm, × 20 magnification. **b**–**e** Typical immunohistochemical labeling for SMAD4 in extrahepatic biliary tract cancer. Scale bar = 50 µm, × 400 magnification. **b** Nuclear SMAD4 expression is negative, score = 0.** c **The percentage of nuclear positive SMAD4 is 0–25%, score = 1. **d** The percentage of nuclear positive SMAD4 is 25–50%, score = 2. **e** The percentage of nuclear positive SMAD4 is above 50%, score = 3

Functional SMAD4 status was evaluated based on the intensity of nuclear staining. We defined ‘negative; score = 0’ when nuclear SMAD4 expression was none, and ‘weakly positive; score = 1’ when the percentage of nuclear positive SMAD4 expression was 0–25%, ‘moderately positive; score = 2’ when the percentage of nuclear positive SMAD4 expression was 25–50% and ‘strongly positive; score = 3’ when the percentage of nuclear positive SMAD4 expression was above 50%. We confirmed the cancer area with hematoxylin and eosin staining of the specimens. The invasion front was defined as the front edge between tumor cells and stromal cells, and the central lesion was defined as the central part of the tumor mass or the tissue near the bile duct lumen (Fig. 2a). The nuclear staining intensity of each slide was scored separately at the invasion front and central lesion. The total score was calculated in the sum of four different 400-fold visual fields. Therefore, the highest score was 12 points, and the lowest score was 0 point. Each slide was evaluated in a blinded manner by two authors (S.K. and H.T.) who did not have any clinical or pathological information regarding the sample in order to avoid bias and subjective interpretation.

### Statistical analysis

All data were expressed as mean ± standard deviation (SD). Between group differences in clinicopathological characteristics were analyzed using Student’s *t* test for continuous variables and chi-squared test for non-continuous variables. Overall survival (OS) and recurrence-free survival (RFS) rates were calculated using the Kaplan–Meier method. Univariate and multivariate analyses were performed using the Cox proportional hazards regression model. *P* < 0.05 was considered significant. All statistical analyses were performed in JMP 14.0 software (SAS Institute, Cary, NC) by Hirotoshi Takayama.

## Results

### Patient characteristics

The entire cohort of 98 eBDC patients is summarized in Table 1. This cohort includes 69 men (70.5%) and 29 women (29.5%). The mean age was 68.2 ± 9.0 years. The main tumor locations were the distal bile duct (*n* = 54, 55.1%) and perihilar bile duct (*n* = 44, 44.9%). Among the included patients, 78 (79.6%) achieved R0 resection, and the other 20 patients (20.4%) had microscopically positive surgical margins (R1 resection). Forty-six patients (46.9%) received adjuvant chemotherapy.

**Table 1:** Clinicopathological parameters of 98 patients with extrahepatic bile duct cancer

**Variables**		***n* (%)**
All		98
Race	Asian	98 (100.0%)
	Others	0 (0.0%)
Gender	Men	69 (70.5%)
	Women	29 (29.6%)
Age		68.2 ± 9.0^a^
Operative procedure	Hepatectomy	44 (44.9%)
	Pancreaticoduodenectomy	54 (55.1%)
Location	Perihilar	44 (44.9%)
	Distal	54 (55.1%)
Histological type	Well or moderately	80 (81.6%)
	Poorly	18 (18.4%)
UICC8th_pT	1 or 2	45 (45.9%)
	3 or 4	53 (54.1%)
UICC8th_pN	0	61 (62.2%)
	1 or 2	37 (37.8%)
Microinvasion into lymphatic system	Absent	47 (48.0%)
	Present	51 (52.0%)
Microinvasion into venous system	Absent	69 (70.4%)
	Present	29 (29.6%)
Microinvasion into nervous system	Absent	27 (27.6%)
	Present	71 (72.4%)
Invasion into liver	Absent	72 (73.5%)
	Present	26 (26.5%)
Invasion into pancreas	Absent	61 (62.2%)
	Present	37 (37.8%)
Invasion into portal vein	Absent	87 (88.8%)
	Present	11 (11.2%)
Invasion into artery	Absent	89 (91.8%)
	Present	8 (8.2%)
Residual tumor	R0	78 (79.6%)
	R1	20 (20.4%)
Adjuvant therapy	Done	46 (46.9%)
	Not done	52 (53.1%)

^a^Average ± standard deviation

### Immunohistochemical findings of SMAD4 in eBDC

The typical immunohistochemical expression of SMAD4 is demonstrated in Fig. 2b–e. SMAD4 expression was found in the cytoplasm and/or nucleus of tumor cells. We evaluated SMAD4 staining in the nuclei of tumor cells as functional SMAD4. Supplemental Figure 1 shows a histogram of the SMAD4 immunohistochemical score at the central lesion and the invasion front of resected specimens. The median SMAD4 staining score was 6 at both the central lesion and invasion front, but the mean ± SD score was 6.13 ± 3.63 at the central lesion and 5.57 ± 3.75 at the invasion front. We defined a score ≤ 6 points as low SMAD4 function and a score ≥ 7 points as high SMAD4 function for both sites.

### Association between functional SMAD4 expression and clinicopathological factors among eBDC patients who underwent upfront surgery

**Fig. 3:**
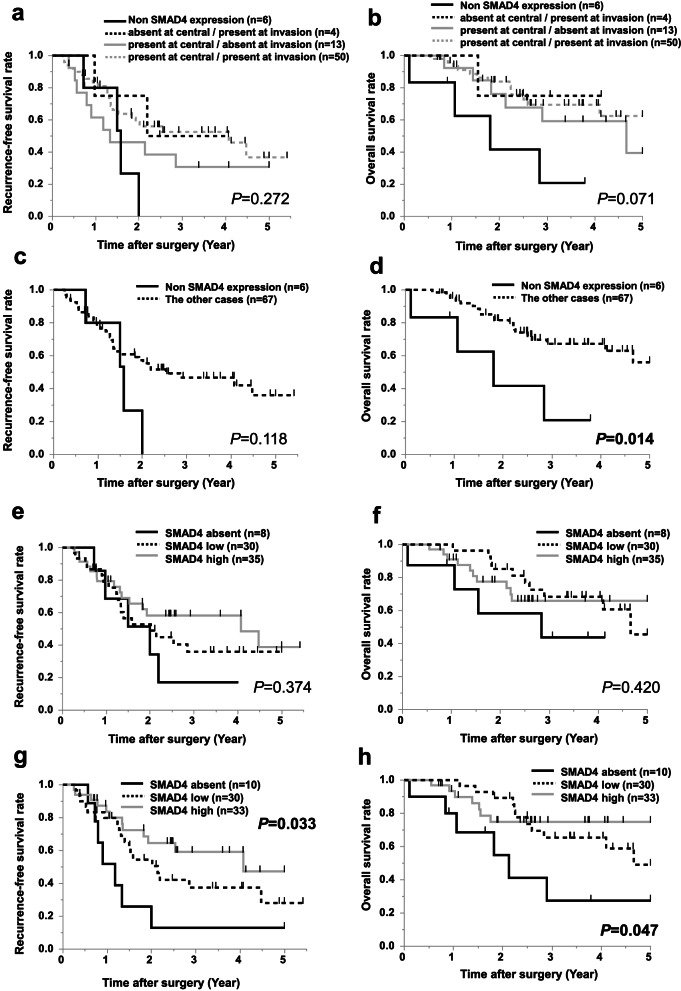
Kaplan–Meier survival curves for 73 patients with upfront surgery stratified by SMAD4 status. **a** Recurrence-free survival. **b** Overall survival. Black solid line, cases without SMAD4 expression at any site (*n* = 6); black dashed line, cases with absent at the central lesion and present at the invasion front (*n* = 4); gray solid line, cases with present at the central lesion and absent at the invasion front (*n* = 13); and gray dashed line, cases with present at both sites (*n* = 50). **c** Recurrence-free survival and **d** Overall survival in dichotomous groups. Black solid line, cases without SMAD4 expression at any site (*n* = 6); black dashed line, the other cases (*n* = 67). **e**–**h** Patients were classified into three groups according to SMAD4 status at the central lesion and invasion front. SMAD4 score is 0 points in the absent group, 1 to 6 points in the low group, and 7 to 12 points in the high group. **e** Recurrence-free survival at the central lesion. **f** Overall survival at the central lesion. **g** Recurrence-free survival at the invasion front. **h** Overall survival at the invasion front

Table 2 shows the correlation between functional SMAD4 staining at the central lesion and tumor invasion front, and Fig. 3a, b shows the RFS and OS curves for four groups created when stratifying by SMAD4 staining at the two sites. There was no significant difference in the analyses, but in the analysis of OS, there was a marginal difference; the group without SMAD4 expression at any site had the poorest prognosis. Next, based on SMAD4 expression at the central lesion and invasion front, we classified upfront surgery patients into two groups: no SMAD4 expression at any site (*n* = 6) and other cases (*n* = 67). Supplemental Table 1 summarizes the comparison of patient characteristics between the two groups. No SMAD4 expression was non-significantly associated with higher invasion of the liver (66.7% vs. 26.9% *P* = 0.053) and nervous system (100.0% vs. 76.9% *P* = 0.078). We did not observe a significant correlation of SMAD4 status with other clinicopathological factors.

**Table 2:** The correlation of SMAD4 expression at tumor central lesion and at tumor invasion front

SMAD4 expression at central lesion	SMAD4 expression at invasion front	
	Absent	Present
Absent	6 (8.2%)	4 (5.5%)
Present	13 (17.8%)	50 (68.5%)

### Association between SMAD4 expression and prognosis among eBDC patients who underwent upfront surgery

Figure 3c, d shows the Kaplan–Meier survival curves for RFS and OS according to SMAD4 expression. Patients without SMAD4 expression at any site had significantly poorer OS than the other patients (3-year OS rate: no SMAD4 expression, 20.83%; other cases, 67.22%; *P* = 0.014). There was no significant difference for RFS (3-year OS rate: no SMAD4 expression, 0.0%; other cases, 46.69%; *P* = 0.120).

### Association between intensity of SMAD4 staining at each area and clinicopathological factors in the upfront surgery group with SMAD4 expression at any site

In 67 cases with SMAD4 expression at any site, the association of prognosis and clinicopathological factors with SMAD4 intensity was evaluated separately for the tumor invasion front and the central lesion. The cases were divided into a SMAD4 low group and SMAD4 high group. Supplemental Table 2 summarizes the association between SMAD4 staining and clinicopathological factors. Cases with low SMAD4 staining at the invasion front presented higher invasion of the venous system (*P* = 0.044) and nervous system (*P* = 0.031). Supplemental Figure 2 shows the survival analysis. We did not find a significant difference between the two groups at both sites.

### Three groups according to SMAD4 status

We divided the 73 patients who underwent upfront surgery into three groups depending on the SMAD4 status in each area. The SMAD4 immunohistochemical score was 0 in the absent group, 1–6 in the low group, and 7–12 in the high group. Supplemental Tables 3 and 4 compare clinicopathological factors among the three groups. In the classification of the central lesion, we found no significant difference in clinicopathological factors among the three groups. In classification of the invasion front, we found a significant difference in microinvasion into the venous system and liver. We also compared the prognosis of the three groups. Figure 3e–h shows the Kaplan–Meier survival curves for RFS and OS. In the analysis of the central lesion, we found no significant difference among the three groups. In contrast, in the analysis on the invasion front, we found a significant difference (RFS *P* = 0.033; OS *P* = 0.047) among the three groups, and the absent group had the shortest RFS and OS.

### SMAD4 expression at the metastatic lymph node

**Fig. 4:**
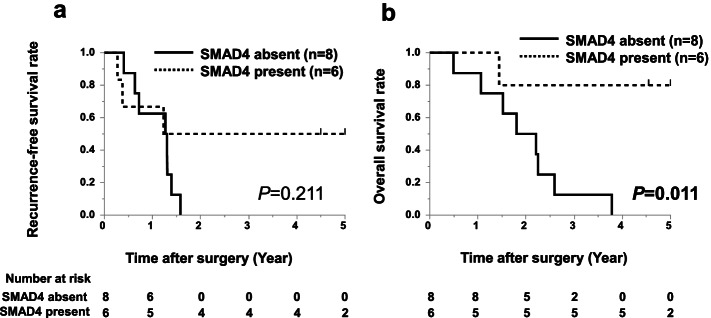
Kaplan–Meier survival curves for 14 patients with lymph node metastasis. **a** Recurrence-free survival stratified by SMAD4 expression at the metastatic lymph node. **b** Overall survival stratified by SMAD4 expression at the metastatic lymph node. Black line, SMAD4 absent group; dashed line, SMAD4 present group

SMAD4 immunostaining of the metastatic lymph node was also performed in 14 cases for which a resected specimen was available. We divided the 14 patients into 6 cases with SMAD4 expression and 8 cases without SMAD4 expression. Figure 4 shows the Kaplan–Meier survival curve stratified by SMAD4 expression in metastatic lymph node. In the RFS analysis, we found no significant difference. In the OS analysis, the group without SMAD4 expression at the metastatic lymph node had a poorer prognosis (*P* = 0.011).

### Association between SMAD4 expression and adjuvant chemotherapy

We also evaluated the correlation between the effect of adjuvant chemotherapy and SMAD4 expression. Supplemental Figure 3 presents the Kaplan–Meier survival curves of 73 patients who underwent upfront surgery stratified by the presence or absence of adjuvant chemotherapy. In the analysis of both RFS and OS, adjuvant chemotherapy did not improve the prognosis. Notably, there was no clear evidence of an effect of adjuvant chemotherapy on eBDC. Next, we classified patients into subgroups depending on the SMAD4 status (low or high) in each area (central lesion and invasion front) and investigated the effect of adjuvant chemotherapy on the prognosis of each group. Supplemental Figures 4 and 5 show the Kaplan–Meier survival curves of these subgroups stratified by the presence or absence of adjuvant chemotherapy. An improving effect of adjuvant chemotherapy was not observed in any subgroup.

### Univariate and multivariate analysis of survival in the upfront surgery group

Supplemental Tables 5 and 6 present the results of univariate and multivariate analyses of factors influencing survival using the Cox proportional hazards model. In the analysis of RFS, univariate analysis showed that invasion into the venous system (hazard ratio [HR] 2.034, *P* = 0.037), invasion of the nervous system (HR 4.711, *P* = 0.011), positive lymph node metastasis (HR 2.152, *P* = 0.024), and residual tumor (HR 2.950, *P* = 0.002) were associated with poor prognosis. Multivariate analysis revealed that invasion of the nervous system (HR 4.250, *P* = 0.023) and residual tumor (HR 2.860, *P* = 0.025) were independent prognostic factors. In the analysis of OS, univariate analysis showed that no SMAD4 expression at any site (HR 3.551, *P* = 0.022), invasion into the lymphatic system (HR 3.136, *P* = 0.013), and invasion of the nervous system (HR 5.269, *P* = 0.024) were prognostic factors. Multivariate analysis revealed that invasion into the lymphatic system (HR 3.136 *P* = 0.024) and invasion of the nervous system (HR 4.606, *P* = 0.043) were independent prognostic factors.

### SMAD4 expression and survival after recurrence

To evaluate the association between SMAD4 and chemotherapy, we also evaluated survival time after recurrence. We consider that the recurrence site would have the same features of the invasion front.

**Fig. 5:**
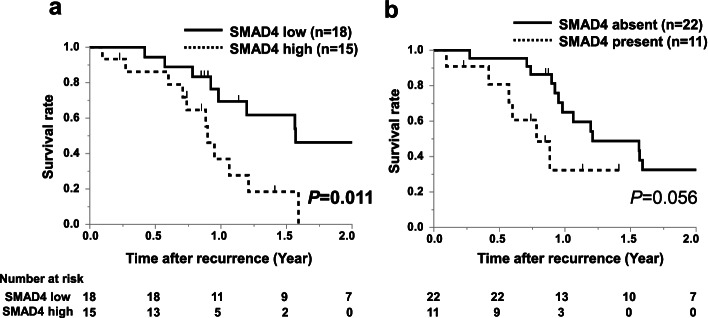
Kaplan–Meier survival curves for 33 patients with recurrence after surgery. **a** Survival curves stratified by SMAD4 expression at the central lesion and **b** the invasion front. Solid line, SMAD4 low expression; dashed line, SMAD4 high expression

Among the 67 patients with SMAD4 expression at any site, 33 experienced recurrence. Survival time after recurrence was evaluated according to SMAD4 status at each central lesion and tumor invasion front (Fig. 5). In both the central lesion and invasion front, the high SMAD4 group had a poorer prognosis than the low SMAD4 group (central lesion, *P* = 0.011; invasion front, *P* = 0.056).

### Change in SMAD4 expression after neoadjuvant chemotherapy

**Fig. 6:**
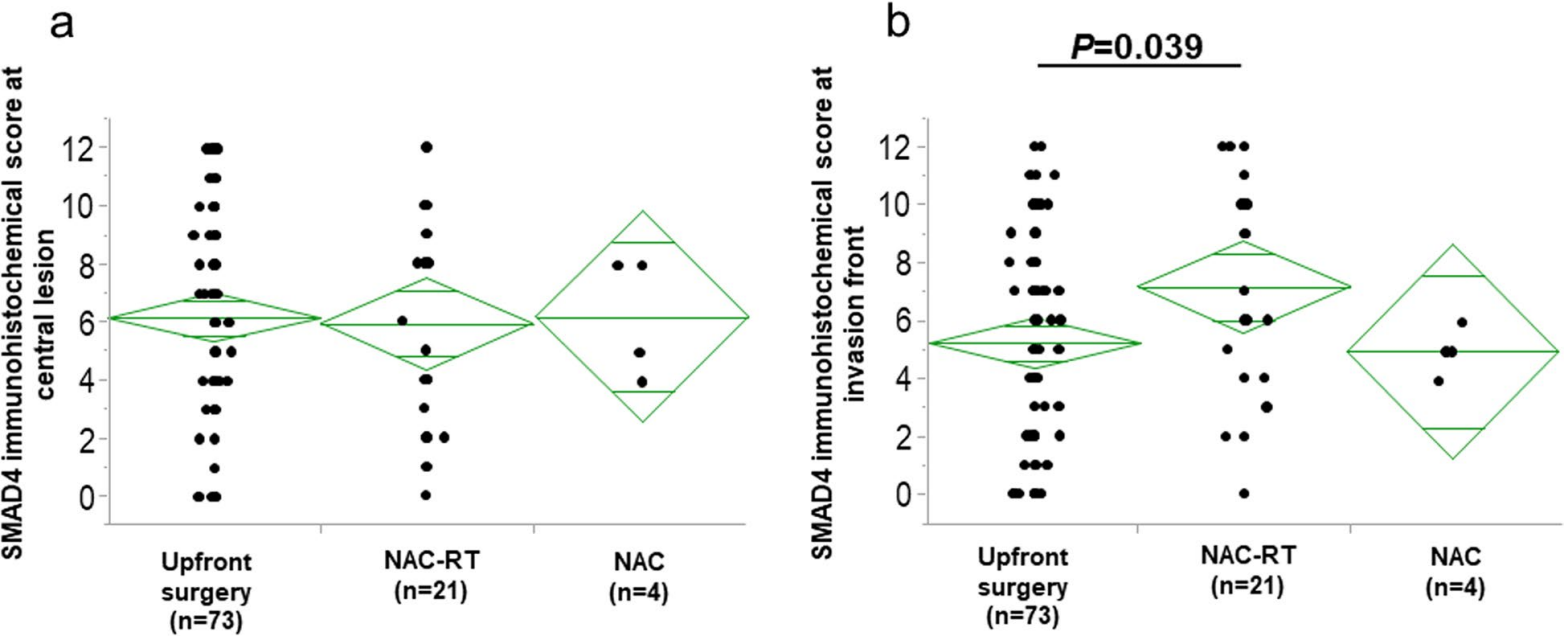
Comparison of SMAD4 immunohistochemical scores among the upfront surgery, NAC and NAC-RT group. **a** At the central lesion. **b** At the invasion front

Finally, to evaluate SMAD4 on the residual cancer after chemotherapy and chemo-radiation therapy, we examined functional SMAD4 in resected specimens after neoadjuvant therapy. Four cases were treated with neoadjuvant chemotherapy (NAC) and 21 cases with neoadjuvant chemo-radiation therapy (NAC-RT), and 73 cases underwent upfront surgery. The clinicopathological factors among the three groups are summarized in Supplemental Table 7. The survival analysis of the three groups is shown in Supplemental Figure 6. The NAC-RT group had better prognosis (5-year RFS rate, 82.4%; 5-year OS rate, 92.9%) than the other groups. Figure 6 shows the SMAD4 immunohistochemical scores at the central lesion and invasion front in each group. At the central lesion, there was no significant difference among the three groups. At the invasion front, we identified a significant difference (*P* = 0.039) between NAC-RT (average score 7.14 ± 3.51) and upfront surgery (average score 5.23 ± 3.75).

## Discussion

SMAD4 is an intracellular transcriptional mediator of the TGFβ signaling pathway. SMAD4 has been reported to function mainly as a tumor suppressor through cell cycle arrest, apoptosis, and differentiation. However, SMAD4 has also been reported to trigger EMT through the induction of EMT-associated transcriptional factors Snail [18], ZEB1, and ZEB2 [19], or to function as a tumor promoter. Along with *KRAS* and *p53*,* SMAD4* is one of the most frequently mutated genes in eBDC [9]. To understand the molecular pathology of BTCs, it is indispensable to elucidate the function of SMAD4. Our previous studies demonstrated high SMAD4 and N-cadherin staining at the invasion front in resected BTC specimens and suggested that EMT may be induced at the cancer invasion front [16]. Our objective was to analyze the intra-tumoral localization of SMAD4 in resected BTC specimens and determine the significance of SMAD4 localization using immunohistochemistry.

We demonstrated that patients without SMAD4 expression at the central lesion or tumor invasion front have poorer OS than those with SMAD4 expression at any site. Furthermore, when patients were classified into three groups (absent, low, high) according to the intensity of functional SMAD4 in each area, the absent group had the poorest prognosis in regards to RFS and OS. In addition, patients whose SMAD4 expression in the metastatic lymph node was negative had poorer OS than SMAD4-positive patients. On the other hand, among patients with recurrence, high SMAD4 expression was significantly related to shorter survival time after recurrence. Moreover, the SMAD4 immunohistochemical score at the tumor invasion front in the group treated with chemo-radiation therapy was lower than the score in the upfront surgery group.

The data presented here demonstrate that the loss of SMAD4 immunostaining was a poor prognostic factor for OS in the upfront surgery group. This result may indicate that, in eBDC, which can be radically resected, SMAD4 inactivation results in a biologically more aggressive form. Previous studies reported that patients without SMAD4 protein expression had a shorter survival time from pancreatic cancer [14], colon cancer [15], and esophageal cancer [20]. In studies of SMAD4 in biliary tract cancer, organ-specific disruption of SMAD4 was shown to induce tumorigenesis of cholangiocarcinoma [21, 22]. Moreover, mutation of the SMAD4 gene was a poor prognostic factor in intrahepatic BDC and the loss of SMAD4 according to immunohistochemistry was significantly associated with distant metastasis [23]. The present result was consistent with these previous reports.

Among cases with recurrence, cases with high SMAD4 expression at any site had shorter survival time than those with low SMAD4 expression. This result may possibly indicate that high expression of functional SMAD4 induces chemoresistance or tumor cells with high SMAD4 expression are more aggressive malignancies when the tumor has progressed to distant metastasis. However, to make such an interpretation, we need to hypothesize that the SMAD4 function at the invasion front in resected specimens is almost the same as its function in recurrent tumors. No reports have examined the prognosis after recurrence in BTCs stratified by the SMAD4 immunostaining status. However, other reports have shown that, in advanced tumors, intact SMAD4 facilitates EMT and TGFβ-dependent tumor growth [24]. We previously found that SMAD4 expression levels are enhanced in the gemcitabine-resistant BDC cell line MzChA-1_GR compared to the parent MzChA-1 cell line, and MzChA-1_GR cells acquired malignant potential through EMT depending on SMAD4 function [16]. It has also been reported that for patients with advanced pancreatic cancer who undergo palliative chemotherapy before resection, patients with preserved SMAD4 expression have significantly shorter progression-free survival than patients with lost SMAD4 expression [25]. TGFβ signaling has also been suggested to play a tumor suppressor role in early-stage tumors but a tumor promotor role in late-stage tumors. Our results may reflect this theory from previous reports.

Functional SMAD4 was expressed at higher levels at the invasion front in patients treated with NAC-RT than in those treated with upfront surgery. OS and RFS were highest in the NAC-RT compared to the other two treatment groups. This result may indicate that tumor cells with low functional SMAD4 expression died by receiving chemo-radiation therapy and tumor cells with high functional SMAD4 expression survived. In short, tumor cells with low functional SMAD4 expression may be radio-sensitive. There have been no reports regarding the evaluation of functional SMAD4 in resected specimens for chemo- and/or radio-sensitivity, and we could not find a report supporting our results. Regarding mutation (deletion) of SMAD4, colorectal cancer [26] and pancreatic ductal carcinoma [27] are associated with resistance to radiotherapy. To examine our results in more detail, we need to compare biopsy specimens taken before neoadjuvant therapy to resected specimens taken after neoadjuvant therapy.

We acknowledge that the present analysis has several limitations. First, this was a retrospective analysis at two institutions and included a small number of patients. Second, the evaluation only by immunohistochemistry is thought to be insufficient to evaluate the presence of SMAD4 expression. Therefore, we have to evaluate SMAD4 expression by other methods, such as Western blotting and PCR. Finally, the present evaluation method for immunostaining cannot cover all areas in a tumor. In the near future, an imaging analysis using high-performance software is needed in order to reflect the exact immunostaining.

In summary, the present results demonstrate that loss of SMAD4 expression is a poor prognostic factor in resectable eBDC and high expression of functional SMAD4 is a poor prognostic factor in recurrent eBDC. These results may indicate that SMAD4 had a bidirectional function as both a tumor promotor and tumor suppressor. In the presence of SMAD4 deletion, pathways other than TGF/SMAD would work in cancer promotion. The function of SMAD4 was complicated and we could not explain the function of SMAD4 completely. In the near future, further investigations of the dual function of SMAD4 are needed.

## Conclusions

The loss of SMAD4 protein expression was a poor prognostic factor in eBDC. The intensity of functional SMAD4 staining in eBDC is a marker of resistance to chemotherapy and radiotherapy. The localization of functional SMAD4 plays a complicated role in eBDC that related not only to the natural course of BTC after surgery, but also chemo-radio-sensitivity.

## Supplementary Information

**Additional file 1: Supplemental Figure 1.** Histogram of the SMAD4 immunohistochemical score for resected specimens. **Supplemental Figure 2.** Kaplan-Meier survival curves for 67 patients with SMAD4 expression at either the central lesion or invasion front stratified by the SMAD4 status in each area. **Supplemental Figure 3.** Kaplan-Meier survival curves for 73 patients who underwent upfront surgery stratified by treatment with adjuvant chemotherapy. **Supplemental Figure 4.** Kaplan-Meier survival curves for patients who underwent upfront surgery stratified by treatment with adjuvant chemotherapy. **Supplemental Figure 5.** Kaplan-Meier survival curves for patients who underwent upfront surgery stratified by treatment with adjuvant chemotherapy. **Supplemental Figure 6.** Kaplan-Meier survival curves for 98 patients stratified by neoadjuvant treatment.

**Additional file 2: Supplemental Table 1.** Association between clinicopathological factors and SMAD4 expression. **Supplemental Table 2.** Association between SMAD4 expression at each area and clinicopathological factors among 67 patients except cases without SMAD4 expression at any site. **Supplemental Table 3.** Association between clinicopathological factors and SMAD4 expression at central lesion. **Supplemental Table 4.** Association between clinicopathological factors and SMAD4 expression at invasion front. **Supplemental Table 5.** Univariate and Multivariate analysis for recurrence free survival of 73 patients with upfront surgery group. **Supplemental Table 6.** Univariate and Multivariate analysis for overall survival of 73 patients with upfront-surgery group. **Supplemental Table 7.** Association between clinicopathological factors and neoadjuvant treatment.

## Data Availability

The datasets used and/or analyzed during the current study are available from the corresponding author on reasonable request.

## Abbreviations

BTC: Biliary tract cancer
BDC: Biliary duct cancer
NAC: Neoadjuvant chemotherapy
NAC-RT: Neoadjuvant chemo-radiation therapy
EMT: Epithelial mesenchymal transition
OS: Overall survival
RFS: Recurrence-free survival

## References

[1] Valle JW (2010). Advances in the treatment of metastatic or unresectable biliary tract cancer. Ann Oncol.

[2] Wang YZ, Li J, Xia Y (2013). Prognostic nomogram for intrahepatic cholangiocarcinoma after partial hepatectomy. J Clin Oncol.

[3] Wang SJ, Lemieux A, Kalpathy-Cramer J (2011). Nomogram for predicting the benefit of adjuvant chemoradiotherapy for resected gallbladder cancer. J Clin Oncol.

[4] Valle J, Wasan H, Palmer DH (2010). Cisplatin plus gemcitabine versus gemcitabine for biliary tract cancer. N Engl J Med.

[5] Okusaka T, Nakachi K, Fukutomi A (2010). Gemcitabine alone or in combination with cisplatin in patients with biliary tract cancer: a comparative multicentre study in Japan. Br J Cancer.

[6] Morizane C, Okusaka T, Mizusawa J (2019). Combination gemcitabine plus S-1 versus gemcitabine plus cisplatin for advanced/recurrent biliary tract cancer: the FUGA-BT (JCOG1113) randomized phase III clinical trial. Ann Oncol.

[7] Valle J (2010). Cisplatin plus gemcitabine versus gemcitabine for biliary tract cancer (vol 362, pg 1273, 2010). N Engl J Med.

[8] Jusakul A, Cutcutache I, Yong CH (2017). Whole-genome and epigenomic landscapes of etiologically distinct subtypes of cholangiocarcinoma. Article Cancer Discovery.

[9] Nakamura H, Arai Y, Totoki Y (2015). Genomic spectra of biliary tract cancer. Article. Nature Genetics..

[10] Monika R, Satya Vj, Chigurupati R, Roli P, Mridula S, Manoj P. MAP Kinase and mammalian target of rapamycin are main pathways of gallbladder carcinogenesis: Results from bioinformatic analysis of Next Generation Sequencing data from a hospital-based cohort. Europe PMC. 2022. 10.1007/s11033-022-07874-4 36018415

[11] Ruhi Dixit, Manoj Pandey, Monika Rajput, Vijay Kumar Shukla. Unravelling of the comparative transcriptomic profile of gallbladder cancer using mRNA sequencing. Mol Biol Rep. 2022; 49:6395–6403. 10.1007/s11033-022-07448-4 35469389

[12] Montal R, Sia D, Montironi C (2020). Molecular classification and therapeutic targets in extrahepatic cholangiocarcinoma. J Hepatol.

[13] Heldin CH, Miyazono K, tenDijke P (1997). TGF-beta signalling from cell membrane to nucleus through SMAD proteins. Nature.

[14] Tascilar M, Skinner HG, Rosty C (2001). The SMAD4 protein and prognosis of pancreatic ductal adenocarcinoma. Clin Cancer Res.

[15] Alazzouzi H, Alhopuro P, Salovaara R (2005). SMAD4 as a prognostic marker in colorectal cancer. Clin Cancer Res.

[16] Yamada D, Kobayashi S, Wada H (2013). Role of crosstalk between interleukin-6 and transforming growth factor-beta 1 in epithelial-mesenchymal transition and chemoresistance in biliary tract cancer. Eur J Cancer.

[17] Shen W, Tao G-Q, Zhang Y, Cai B, Sun J, Tian Z-Q. TGF-β in pancreatic cancer initiation and progression: two sides of the same coin. Cell Bioscience. 2017;7(39) 10.1186/s13578-017-0168-0 PMC5545849 28794854

[18] Vincent T, Neve EPA, Johnson JR (2009). A SNAIL1-SMAD3/4 transcriptional repressor complex promotes TGF-beta mediated epithelial-mesenchymal transition. Nat Cell Biol.

[19] Gregory PA, Bracken CP, Smith E (2011). An autocrine TGF-beta/ZEB/miR-200 signaling network regulates establishment and maintenance of epithelial-mesenchymal transition. Mol Biol Cell.

[20] Natsugoe S, Che XM, Matsumoto M (2002). Smad4 and transforming growth factor beta 1 expression in patients with squamous cell carcinoma of the esophagus. Clin Cancer Res.

[21] Xu X (2006). Induction of intrahepatic cholangiocellular carcinoma by liver-specific disruption ofSmad4 andPten in mice. J Clin Investig.

[22] Kobayashi S, Werneburg NW, Bronk SF, Kaufmann SH, Gores GJ (2005). Interleukin-6 contributes to Mcl-1 Up-regulation and TRAIL resistance via an Akt-signaling pathway in cholangiocarcinoma cells. Gastroenterology.

[23] Yan XQ, Zhang W, Zhang BX, Liang HF, Zhang WG, Chen XP (2013). Inactivation of Smad4 is a prognostic factor in intrahepatic cholangiocarcinoma. Article Chin Med J.

[24] Ormanns S, Haas M, Remold A (2017). The impact of SMAD4 loss on outcome in patients with advanced pancreatic cancer treated with systemic chemotherapy. Int J Mol Sci.

[25] Bardeesy N, Cheng KH, Berger JH (2006). Smad4 is dispensable for normal pancreas development yet critical in progression and tumor biology of pancreas cancer. Genes Dev.

[26] Jiang D, Wang X, Wang Y (2019). Mutation in BRAF and SMAD4 associated with resistance to neoadjuvant chemoradiation therapy in locally advanced rectal cancer. Virchows Arch.

[27] Wang F, Xia X, Yang C (2018). SMAD4 gene mutation renders pancreatic cancer resistance to radiotherapy through promotion of autophagy. Clin Cancer Res.

