# Supplementary figures and images for: Prognostic value of functional SMAD4 localization in extrahepatic bile duct cancer
Source: World J Surg Oncol. 2022 Sep 10;20:291. doi: 10.1186/s12957-022-02747-3 (PMC9463834; doi:10.1186/s12957-022-02747-3)

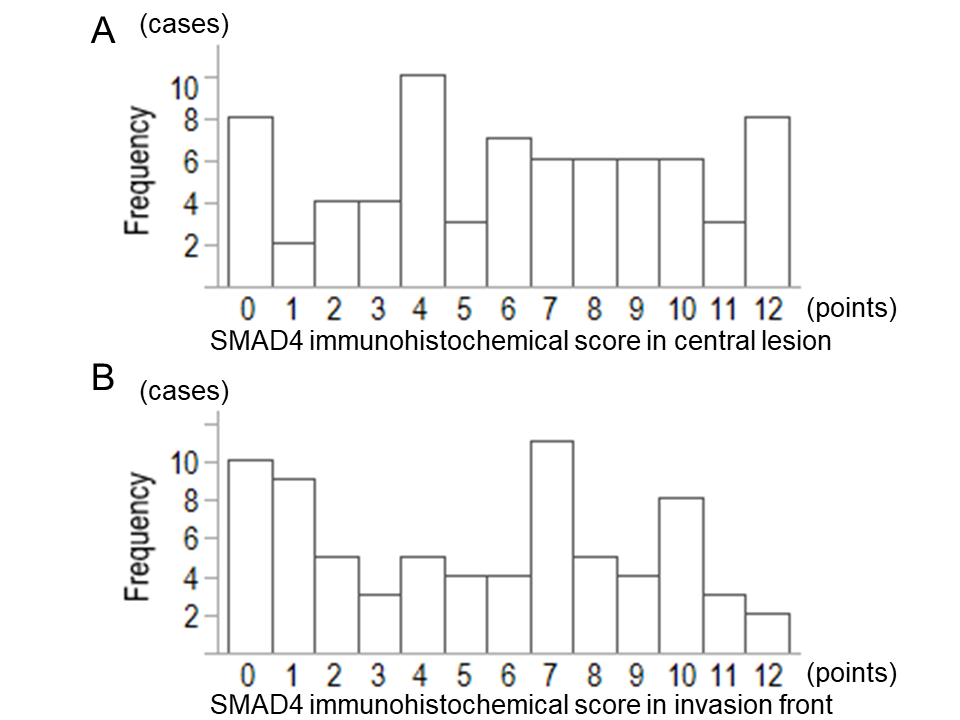

Supplement: Supplementary file 1 — Additional file 1: Supplemental Figure 1. Histogram of the SMAD4 immunohistochemical score for resected specimens. Supplemental Figure 2. Kaplan-Meier survival curves for 67 patients with SMAD4 expression at either the central lesion or invasion front stratified by the SMAD4 status in each area. Supplemental Figure 3. Kaplan-Meier survival curves for 73 patients who underwent upfront surgery stratified by treatment with adjuvant chemotherapy. Supplemental Figure 4. Kaplan-Meier survival curves for patients who underwent upfront surgery stratified by treatment with adjuvant chemotherapy. Supplemental Figure 5. Kaplan-Meier survival curves for patients who underwent upfront surgery stratified by treatment with adjuvant chemotherapy. Supplemental Figure 6. Kaplan-Meier survival curves for 98 patients stratified by neoadjuvant treatment. [file 12957_2022_2747_MOESM1_ESM.zip › Supplemental Figure1_revised.tif]

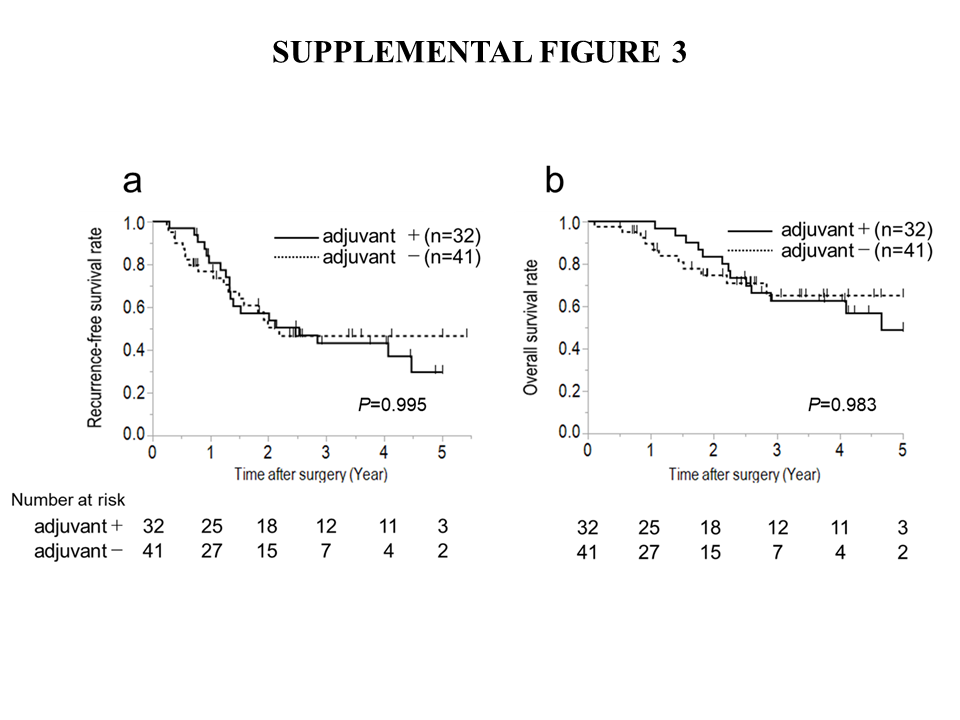

Supplement: Supplementary file 1 — Additional file 1: Supplemental Figure 1. Histogram of the SMAD4 immunohistochemical score for resected specimens. Supplemental Figure 2. Kaplan-Meier survival curves for 67 patients with SMAD4 expression at either the central lesion or invasion front stratified by the SMAD4 status in each area. Supplemental Figure 3. Kaplan-Meier survival curves for 73 patients who underwent upfront surgery stratified by treatment with adjuvant chemotherapy. Supplemental Figure 4. Kaplan-Meier survival curves for patients who underwent upfront surgery stratified by treatment with adjuvant chemotherapy. Supplemental Figure 5. Kaplan-Meier survival curves for patients who underwent upfront surgery stratified by treatment with adjuvant chemotherapy. Supplemental Figure 6. Kaplan-Meier survival curves for 98 patients stratified by neoadjuvant treatment. [file 12957_2022_2747_MOESM1_ESM.zip › supplemental figure3.TIF]

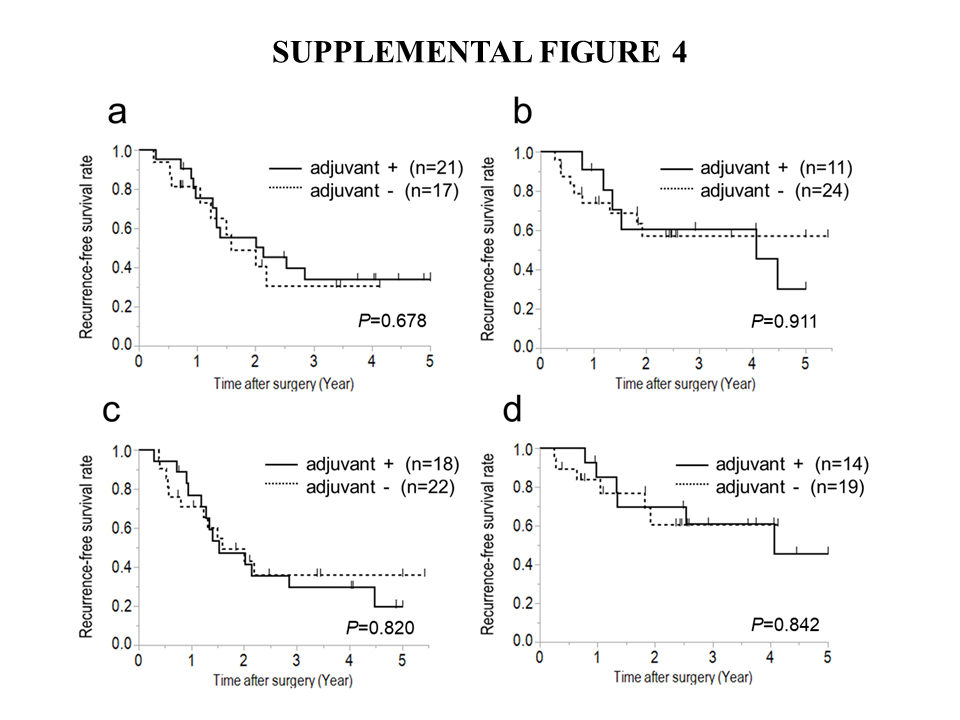

Supplement: Supplementary file 1 — Additional file 1: Supplemental Figure 1. Histogram of the SMAD4 immunohistochemical score for resected specimens. Supplemental Figure 2. Kaplan-Meier survival curves for 67 patients with SMAD4 expression at either the central lesion or invasion front stratified by the SMAD4 status in each area. Supplemental Figure 3. Kaplan-Meier survival curves for 73 patients who underwent upfront surgery stratified by treatment with adjuvant chemotherapy. Supplemental Figure 4. Kaplan-Meier survival curves for patients who underwent upfront surgery stratified by treatment with adjuvant chemotherapy. Supplemental Figure 5. Kaplan-Meier survival curves for patients who underwent upfront surgery stratified by treatment with adjuvant chemotherapy. Supplemental Figure 6. Kaplan-Meier survival curves for 98 patients stratified by neoadjuvant treatment. [file 12957_2022_2747_MOESM1_ESM.zip › supplemental figure4.TIF]

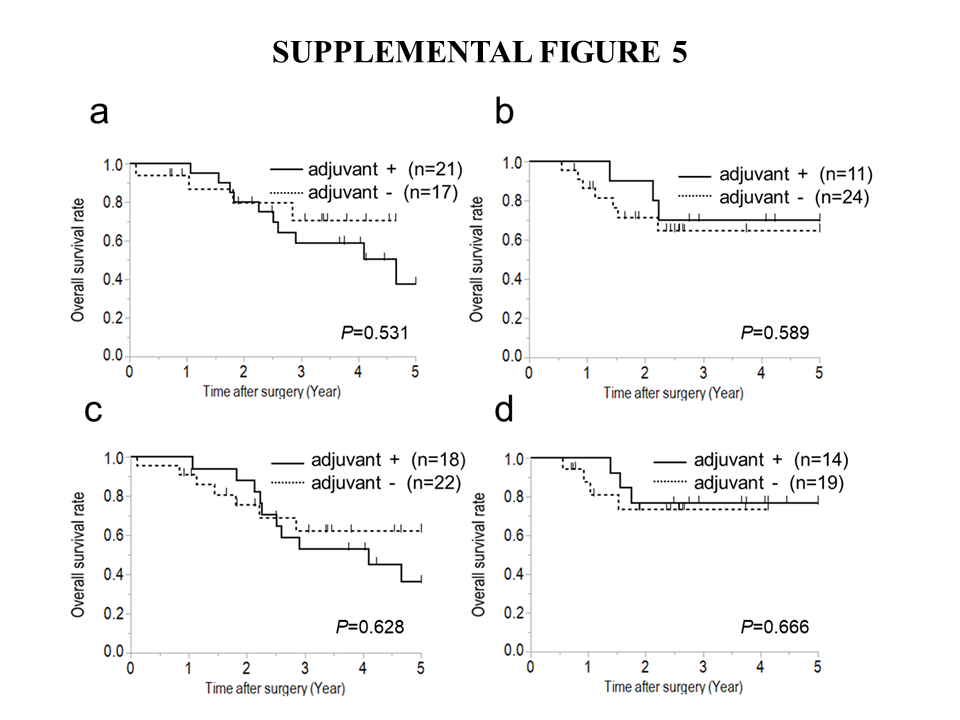

Supplement: Supplementary file 1 — Additional file 1: Supplemental Figure 1. Histogram of the SMAD4 immunohistochemical score for resected specimens. Supplemental Figure 2. Kaplan-Meier survival curves for 67 patients with SMAD4 expression at either the central lesion or invasion front stratified by the SMAD4 status in each area. Supplemental Figure 3. Kaplan-Meier survival curves for 73 patients who underwent upfront surgery stratified by treatment with adjuvant chemotherapy. Supplemental Figure 4. Kaplan-Meier survival curves for patients who underwent upfront surgery stratified by treatment with adjuvant chemotherapy. Supplemental Figure 5. Kaplan-Meier survival curves for patients who underwent upfront surgery stratified by treatment with adjuvant chemotherapy. Supplemental Figure 6. Kaplan-Meier survival curves for 98 patients stratified by neoadjuvant treatment. [file 12957_2022_2747_MOESM1_ESM.zip › supplemental figure5.TIF]

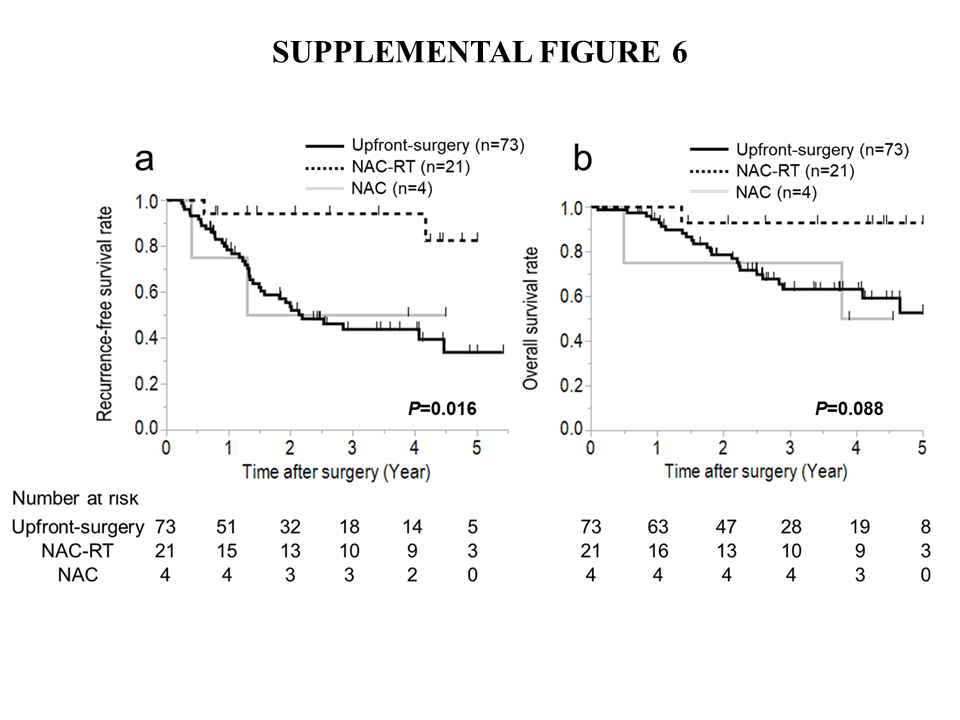

Supplement: Supplementary file 1 — Additional file 1: Supplemental Figure 1. Histogram of the SMAD4 immunohistochemical score for resected specimens. Supplemental Figure 2. Kaplan-Meier survival curves for 67 patients with SMAD4 expression at either the central lesion or invasion front stratified by the SMAD4 status in each area. Supplemental Figure 3. Kaplan-Meier survival curves for 73 patients who underwent upfront surgery stratified by treatment with adjuvant chemotherapy. Supplemental Figure 4. Kaplan-Meier survival curves for patients who underwent upfront surgery stratified by treatment with adjuvant chemotherapy. Supplemental Figure 5. Kaplan-Meier survival curves for patients who underwent upfront surgery stratified by treatment with adjuvant chemotherapy. Supplemental Figure 6. Kaplan-Meier survival curves for 98 patients stratified by neoadjuvant treatment. [file 12957_2022_2747_MOESM1_ESM.zip › supplemental figure6.TIF]

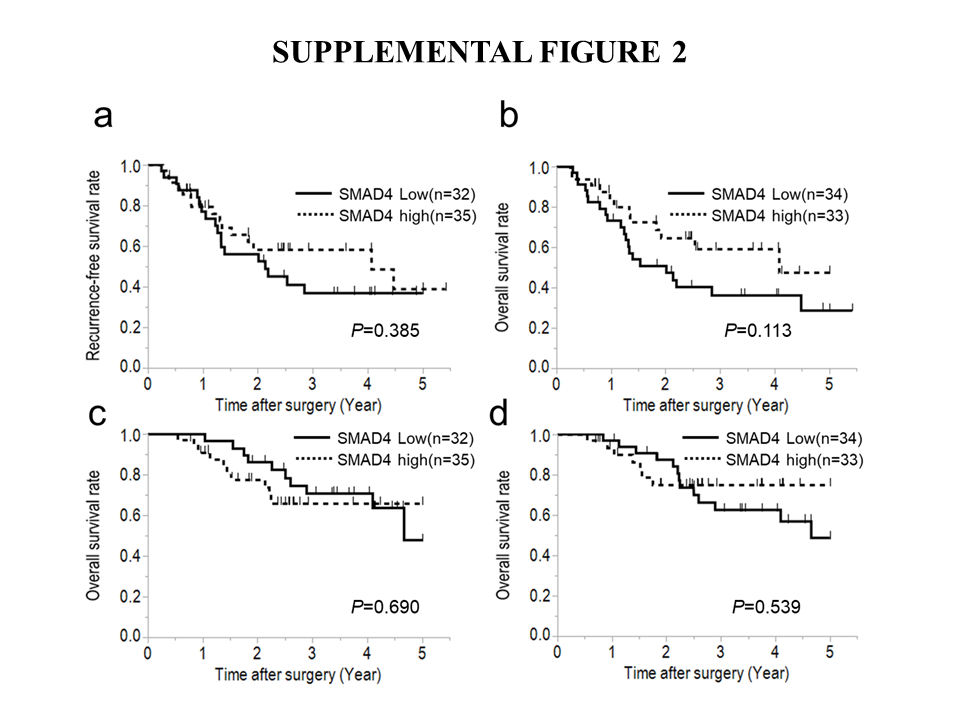

Supplement: Supplementary file 1 — Additional file 1: Supplemental Figure 1. Histogram of the SMAD4 immunohistochemical score for resected specimens. Supplemental Figure 2. Kaplan-Meier survival curves for 67 patients with SMAD4 expression at either the central lesion or invasion front stratified by the SMAD4 status in each area. Supplemental Figure 3. Kaplan-Meier survival curves for 73 patients who underwent upfront surgery stratified by treatment with adjuvant chemotherapy. Supplemental Figure 4. Kaplan-Meier survival curves for patients who underwent upfront surgery stratified by treatment with adjuvant chemotherapy. Supplemental Figure 5. Kaplan-Meier survival curves for patients who underwent upfront surgery stratified by treatment with adjuvant chemotherapy. Supplemental Figure 6. Kaplan-Meier survival curves for 98 patients stratified by neoadjuvant treatment. [file 12957_2022_2747_MOESM1_ESM.zip › supplemnetal figure2.TIF]
