# Supplementary material for: Prognostic value of functional SMAD4 localization in extrahepatic bile duct cancer
Source: World J Surg Oncol. 2022 Sep 10;20:291. doi: 10.1186/s12957-022-02747-3 (PMC9463834; doi:10.1186/s12957-022-02747-3)
Supplement: Supplementary file 2 — Additional file 2: Supplemental Table 1. Association between clinicopathological factors and SMAD4 expression. Supplemental Table 2. Association between SMAD4 expression at each area and clinicopathological factors among 67 patients except cases without SMAD4 expression at any site. Supplemental Table 3. Association between clinicopathological factors and SMAD4 expression at central lesion. Supplemental Table 4. Association between clinicopathological factors and SMAD4 expression at invasion front. Supplemental Table 5. Univariate and Multivariate analysis for recurrence free survival of 73 patients with upfront surgery group. Supplemental Table 6. Univariate and Multivariate analysis for overall survival of 73 patients with upfront-surgery group. Supplemental Table 7. Association between clinicopathological factors and neoadjuvant treatment. [file 12957_2022_2747_MOESM2_ESM.docx]

| **SUPPLEMENTAL TABLE 1**  Association between clinicopathological factors and SMAD4 expression. | | | | | |  |
| --- | --- | --- | --- | --- | --- | --- |
|  |  |  |  |  |  |  |
|  |  | **SMAD4 expression** | | | |  |
| **Variables** |  | **No expression at any site*^1^** |  | **The other cases***^2^ | ***P value*** |  |
| All |  | 6 (100.0%) |  | 67 (100.0%) |  |  |
| Gender | Men | 4 (66.7%) |  | 48 (71.6%) | 0.799 |  |
|  | Women | 2 (33.3%) |  | 19 (28.4%) |  |  |
| Age |  | 74.2±3.9^＊3^ |  | 69.4±8.0^＊3^ | 0.114 |  |
| Operative procedure | Hepatectomy | 5 (83.3%) |  | 31 (46.3%) | 0.071 |  |
|  | Pancreaticoduodenectomy | 1 (16.7%) |  | 36 (53.7%) |  |  |
| Location | Perihilar | 5 (83.3%) |  | 31 (46.3%) | 0.071 |  |
|  | Distal | 1 (16.7%) |  | 36 (53.7%) |  |  |
| Histological type | Well or moderately | 6 (100.0%) |  | 53 (79.1%) | 0.102 |  |
|  | Poorly | 0 (0.0%) |  | 14 (20.9%) |  |  |
| UICC8th_pT | 1 or 2 | 4 (66.7%) |  | 29 (43.3%) | 0.269 |  |
|  | 3 or 4 | 2 (33.3%) |  | 38 (56.7%) |  |  |
| UICC8th_pN | 0 | 3 (50.0%) |  | 39 (58.2%) | 0.698 |  |
|  | 1 or 2 | 3 (50.0%) |  | 28 (41.8%) |  |  |
| Microinvasion into lymphatic system | Absent | 2 (33.3%) |  | 28 (41.8%) | 0.683 |  |
|  | Present | 4 (66.6%) |  | 39 (58.2%) |  |  |
| Microinvasion into venous system | Absent | 3 (50.0%) |  | 45 (67.2%) | 0.407 |  |
|  | Present | 3 (50.0%) |  | 22 (32.8%) |  |  |
| Microinvasion into nervous system | Absent | 0 (0.0%) |  | 16 (23.9%) | 0.078 |  |
|  | Present | 6 (100.0%) |  | 51 (76.1%) |  |  |
| Invasion into liver | Absent | 2 (33.3%) |  | 49 (73.1%) | 0.053 |  |
|  | Present | 4 (66.7%) |  | 18 (26.9%) |  |  |
| Invasion into pancreas | Absent | 5 (83.3%) |  | 41 (61.2%) | 0.255 |  |
|  | Present | 1 (16.7%) |  | 26 (38.8%) |  |  |
| Invasion into portal vein | Absent | 5 (83.3%) |  | 61 (91.0%) | 0.570 |  |
|  | Present | 1 (16.7%) |  | 6 (9.0%) |  |  |
| Invasion into artery | Absent | 5 (83.3%) |  | 61 (91.0%) | 0.487 |  |
|  | Present | 1 (16.7%) |  | 6 (9.0%) |  |  |
| Residual tumor | R0 | 4 (66.7%) |  | 52 (77.6%) | 0.559 |  |
|  | R1 | 2 (33.3%) |  | 15 (22.4%) |  |  |
| Adjuvant therapy | Done | 1 (16.7%) |  | 31 (46.3%) | 0.141 |  |
|  | Not done | 5 (83.3%) |  | 36 (53.7%) |  |  |
| ＊1:cases without SMAD4 expression at both central lesion and invasion front  ＊2:cases with SMAD4 expression at either central lesion or invasion front  ＊3:average ± standard deviation | | | | | | |

| **SUPPLEMENTAL TABLE 2**  Association between SMAD4 expression at each area and clinicopathological factors among 67 patients except cases without SMAD4 expression at any site. | | | | | | | | | | |
| --- | --- | --- | --- | --- | --- | --- | --- | --- | --- | --- |
|  |  | **SMAD4 expression**  **in central lesion** | | | |  | **SMAD4 expression**  **in invasion front** | | | |
| **Variables** |  | **Low** |  | **High** | ***P value*** |  | **Low** |  | **High** | ***P value*** |
| All |  | 32 |  | 35 |  |  | 34 |  | 33 |  |
| Gender | Men | 20 (62.5%) |  | 28 (80.0%) | 0.111 |  | 22 (64.7%) |  | 26 (78.8%) | 0.199 |
|  | Women | 12 (37.5%) |  | 7 (20.0%) |  |  | 12 (35.3%) |  | 7 (21.2%) |  |
| Age |  | 66.8±9.0^＊^ |  | 71.9±6.0^＊^ | **0.008** |  | 67.8±9.7^＊^ |  | 71.2±5.3^＊^ | 0.084 |
| Operative procedure | Hepatectomy | 17 (53.1%) |  | 14 (40.0%) | 0.281 |  | 17 (50.0%) |  | 14 (42.4%) | 0.534 |
|  | Pancreaticoduodenectomy | 15 (46.9%) |  | 21 (60.0%) |  |  | 17 (50.0%) |  | 19 (57.6%) |  |
| Location | Perihilar | 17 (53.1%) |  | 14 (40.0%) | 0.281 |  | 17 (50.0%) |  | 14 (42.4%) | 0.534 |
|  | Distal | 15 (46.9%) |  | 21 (60.0%) |  |  | 17 (50.0%) |  | 19 (57.6%) |  |
| Histological type | Well or moderately | 25 (78.1%) |  | 28 (80.0%) | 0.851 |  | 27 (79.4%) |  | 26 (78.8%) | 0.950 |
|  | Poorly | 7 (21.9%) |  | 7 (20.0%) |  |  | 7 (20.6%) |  | 7 (21.2%) |  |
| UICC8th_pT | 1 or 2 | 15 (46.9%) |  | 14 (40.0%) | 0.570 |  | 13 (38.2%) |  | 16 (48.5%) | 0.397 |
|  | 3 or 4 | 17 (53.1%) |  | 21 (60.0%) |  |  | 21 (61.8%) |  | 17 (51.5%) |  |
| UICC8th_pN | 0 | 15 (46.9%) |  | 24 (68.6%) | 0.071 |  | 18 (52.9%) |  | 21 (63.6%) | 0.374 |
|  | 1 or 2 | 17 (53.1%) |  | 11 (31.4%) |  |  | 16 (47.1%) |  | 12 (36.4%) |  |
| Microinvasion into lymphatic system | Absent | 13 (40.6%) |  | 15 (42.9%) | 0.769 |  | 13 (38.2%) |  | 15 (45.5%) | 0.549 |
|  | Present | 19 (59.4%) |  | 20 (57.1%) |  |  | 21 (61.8%) |  | 18 (54.5%) |  |
| Microinvasion into venous system | Absent | 21 (65.6%) |  | 24 (64.3%) | 0.798 |  | 19 (55.9%) |  | 26 (78.7%) | **0.044** |
|  | Present | 11 (34.4%) |  | 11 (31.4%) |  |  | 15 (44.1%) |  | 7 (21.2%) |  |
| Microinvasion into nervous system | Absent | 8 (25.0%) |  | 8 (22.9%) | 0.837 |  | 6 (17.7%) |  | 10 (30.3%) | 0.228 |
|  | Present | 24 (75.0%) |  | 27 (77.1%) |  |  | 28 (82.4%) |  | 23 (69.7%) |  |
| Invasion into liver | Absent | 21 (65.6%) |  | 28 (80.0%) | 0.184 |  | 21 (61.8%) |  | 28 (84.8%) | **0.031** |
|  | Present | 11 (34.4%) |  | 7 (20.0%) |  |  | 13 (38.2%) |  | 5 (15.2%) |  |
| Invasion into pancreas | Absent | 21 (65.6%) |  | 20 (57.1%) | 0.476 |  | 22 (64.7%) |  | 19 (57.6%) | 0.549 |
|  | Present | 11 (34.4%) |  | 15 (42.9%) |  |  | 12 (35.3%) |  | 14 (42.4%) |  |
| Invasion into portal vein | Absent | 29 (90.6%) |  | 32 (91.4%) | 0.908 |  | 30 (88.2%) |  | 31 (93.9%) | 0.409 |
|  | Present | 3 (9.4%) |  | 3 (8.6%) |  |  | 4 (11.8%) |  | 2 (6.1%) |  |
| Invasion into artery | Absent | 29 (90.6%) |  | 32 (91.4%) | 0.591 |  | 30 (88.2%) |  | 31 (93.9%) | 0.170 |
|  | Present | 3 (9.4%) |  | 2 (5.7%) |  |  | 4 (11.8%) |  | 1 (3.0%) |  |
| Residual tumor | R0 | 24 (73.7%) |  | 28 (80.0%) | 0.624 |  | 25 (73.5%) |  | 27 (81.8%) | 0.414 |
|  | R1 | 8 (26.3%) |  | 7 (20.0%) |  |  | 9 (26.5%) |  | 6 (1.8%) |  |
| Adjuvant therapy | Done | 20 (62.5%) |  | 11 (31.4%) | **0.010** |  | 17 (50.0%) |  | 14 (42.4%) | 0.534 |
|  | Not done | 12 (37.5%) |  | 24 (68.6%) |  |  | 17 (50.0%) |  | 19 (57.6%) |  |
| ＊average ± standard deviation  P values of <0.05 are indicated in bold |  |  |  |  |  |  |  |  |  |  |

| **SUPLLEMENTAL TABLE 3**  Association between clinicopathological factors and SMAD4 expression at central lesion. | | | | | | |  |
| --- | --- | --- | --- | --- | --- | --- | --- |
|  |  |  |  |  |  |  |  |
| **Variables** |  | **Absent^＊1^** |  | **Low^＊2^** |  | **High^＊3^** | ***P value*** |
| All |  | 8 |  | 30 |  | 35 |  |
| Gender | Men | 7 (87.5%) |  | 17 (56.7%) |  | 28 (80.0%) | 0.064 |
|  | Women | 1 (12.5%) |  | 13 (43.3%) |  | 7 (20.0%) |  |
| Age |  | 72.4±5.8^＊4^ |  | 66.7±9.2^＊4^ |  | 71.9±6.0^＊4^ | **0.016** |
| Operative procedure | Hepatectomy | 6 (75.0%) |  | 16 (53.3%) |  | 14 (40.0%) | 0.164 |
|  | Pancreaticoduodenectomy | 2 (25.0%) |  | 14 (46.7%) |  | 21 (60.0%) |  |
| Location | Perihilar | 6 (75.0%) |  | 16 (53.3%) |  | 14 (40.0%) | 0.164 |
|  | Distal | 2 (25.0%) |  | 14 (46.7%) |  | 21 (60.0%) |  |
| Histological type | Well or Moderately | 6 (75.0%) |  | 25 (83.3%) |  | 28 (80.0%) | 0.859 |
|  | Poorly | 2 (25.0%) |  | 5 (16.7%) |  | 7 (20.0%) |  |
| UICC8th_pT | 1 or 2 | 6 (75.0%) |  | 13 (43.3%) |  | 14 (40.0%) | 0.186 |
|  | 3 or 4 | 2 (25.0%) |  | 17 (56.7%) |  | 21 (60.0%) |  |
| UICC8th_pN | 0 | 4 (50.0%) |  | 14 (46.7%) |  | 24 (68.6%) | 0.181 |
|  | 1 or 2 | 4 (50.0%) |  | 16 (53.3%) |  | 11 (31.4%) |  |
| Microinvasion into lymphatic system | Absent | 4 (50.0%) |  | 11 (36.7%) |  | 15 (42.9%) | 0.760 |
|  | Present | 4 (50.0%) |  | 19 (63.3%) |  | 20 (57.1%) |  |
| Microinvasion into venous system | Absent | 5 (62.5%) |  | 19 (63.3%) |  | 24 (68.6%) | 0.887 |
|  | Present | 3 (37.5%) |  | 11 (36.7%) |  | 11 (31.4%) |  |
| Microinvasion into nervous system | Absent | 2 (25.0%) |  | 6 (20.0%) |  | 8 (22.9%) | 0.939 |
|  | Present | 6 (75.0%) |  | 24 (80.0%) |  | 27 (77.1%) |  |
| Invasion into liver | Absent | 3 (37.5%) |  | 20 (66.7%) |  | 28 (80.0%) | 0.062 |
|  | Present | 5 (62.5%) |  | 10 (33.3%) |  | 7 (20.0%) |  |
| Invasion into pancreas | Absent | 6 (75.0%) |  | 20 (66.7%) |  | 20 (57.1%) | 0.547 |
|  | Present | 2 (25.0%) |  | 10 (33.3%) |  | 15 (42.9%) |  |
| Invasion into portal vein | Absent | 8 (100.0%) |  | 26 (86.7%) |  | 32 (91.4%) | 0.351 |
|  | Present | 0 (0.0%) |  | 4 (13.3%) |  | 3 (5.9%) |  |
| Invasion into artery | Absent | 8 (100.0%) |  | 26 (86.7%) |  | 32 (94.1%) | 0.282 |
|  | Present | 0 (0.0%) |  | 4 (13.3%) |  | 2 (8.3%) |  |
| Residual tumor | R0 | 7 (87.5%) |  | 26 (86.7%) |  | 33 (94.3%) | 0.546 |
|  | R1 | 1 (12.5%) |  | 4 (13.3%) |  | 2 (5.7%) |  |
| Adjuvant therapy | Done | 3 (37.5%) |  | 18 (60.0%) |  | 11 (31.4%) | 0.062 |
|  | Not done | 5 (62.5%) |  | 12 (40.0%) |  | 24 (68.6%) |  |
| ＊1:SMAD4 immunohistochemical score is 0 point  ＊2:SMAD4 immunohistochemical score is 1-6 points  ＊3:SMAD4 immunohistochemical score is 7-12 points  ＊4:average ± standard deviation  P value of <0.05 are indicated in bold | | | | | | | |

| **SUPPLEMENTAL TABLE 4**  Association between clinicopathological factors and SMAD4 expression at invasion front. | | | | | | | |
| --- | --- | --- | --- | --- | --- | --- | --- |
| **Variables** |  | **Absent^＊1^** |  | **Low^＊2^** |  | **High^＊3^** | ***P value*** |
| All |  | 10 |  | 30 |  | 33 |  |
| Gender | Men | 7 (87.5%) |  | 19 (63.3%) |  | 26 (78.8%) | 0.396 |
|  | Women | 3 (12.5%) |  | 11 (36.7%) |  | 7 (21.2%) |  |
| Age |  | 70.8±9.3^＊4^ |  | 68.1±9.4^＊4^ |  | 71.2±5.3^＊4^ | 0.272 |
| Operative procedure | Hepatectomy | 7 (70.0%) |  | 15 (50.0%) |  | 14 (42.4%) | 0.302 |
|  | Pancreaticoduodenectomy | 3 (30.0%) |  | 15 (50.0%) |  | 19 (57.6%) |  |
| Location | Perihilar | 7 (70.0%) |  | 15 (50.0%) |  | 14 (42.4%) | 0.302 |
|  | Distal | 3 (30.0%) |  | 15 (50.0%) |  | 19 (57.6%) |  |
| Histological type | Well or Moderately | 8 (80.0%) |  | 25 (83.3%) |  | 26 (78.8%) | 0.897 |
|  | Poorly | 2 (20.0%) |  | 5 (16.7%) |  | 7 (21.2%) |  |
| UICC8th_pT | 1 or 2 | 5 (50.0%) |  | 12 (40.0%) |  | 16 (48.5%) | 0.754 |
|  | 3 or 4 | 5 (50.0%) |  | 18 (60.0%) |  | 17 (51.5%) |  |
| UICC8th_pN | 0 | 6 (60.0%) |  | 15 (50.0%) |  | 21 (63.6%) | 0.542 |
|  | 1 or 2 | 4 (40.0%) |  | 15 (50.0%) |  | 12 (36.4%) |  |
| Microinvasion into lymphatic system | Absent | 4 (40.0%) |  | 11 (36.7%) |  | 15 (45.5%) | 0.776 |
|  | Present | 6 (60.0%) |  | 19 (63.3%) |  | 18 (54.6%) |  |
| Microinvasion into venous system | Absent | 8 (80.0%) |  | 14 (46.7%) |  | 26 (78.8%) | **0.016** |
|  | Present | 2 (20.0%) |  | 16 (53.3%) |  | 7 (21.2%) |  |
| Microinvasion into nervous system | Absent | 1 (10.0%) |  | 5 (16.7%) |  | 10 (30.3%) | 0.252 |
|  | Present | 9 (90.0%) |  | 25 (83.3%) |  | 23 (69.7%) |  |
| Invasion into liver | Absent | 4 (40.0%) |  | 19 (63.3%) |  | 28 (84.9%) | **0.015** |
|  | Present | 6 (60.0%) |  | 11 (36.7%) |  | 5 (15.2%) |  |
| Invasion into pancreas | Absent | 9 (90.0%) |  | 18 (60.0%) |  | 19 (57.6%) | 0.115 |
|  | Present | 1 (10.0%) |  | 12 (40.0%) |  | 14 (42.4%) |  |
| Invasion into portal vein | Absent | 9 (90.0%) |  | 26 (86.7%) |  | 31 (93.9%) | 0.613 |
|  | Present | 1 (10.0%) |  | 4 (13.3%) |  | 2 (6.1%) |  |
| Invasion into artery | Absent | 9 (90.0%) |  | 26 (86.7%) |  | 31 (96.9%) | 0.310 |
|  | Present | 1 (10.0%) |  | 4 (13.3%) |  | 1 (3.1%) |  |
| Residual tumor | R0 | 8 (80.0%) |  | 27 (90.0%) |  | 31 (93.9%) | 0.466 |
|  | R1 | 2 (20.0%) |  | 3 (10.0%) |  | 2 (6.1%) |  |
| Adjuvant therapy | Done | 4 (40.0%) |  | 14 (46.7%) |  | 14 (42.4%) | 0.912 |
|  | Not done | 6 (60.0%) |  | 16 (53.3%) |  | 19 (57.6%) |  |
| ＊1:SMAD4 immunohistochemical score is 0 point  ＊2:SMAD4 immunohistochemical score is 1-6 points  ＊3:SMAD4 immunohistochemical score is 7-12 points  ＊4:average ± standard deviationP value of <0.05 are indicated in bold | | | | | | | |

| **SUPPLEMENTAL TABLE 5**  Univariate and Multivariate analysis for recurrence free survival of 73 patients with upfront surgery group. | | | | | | |
| --- | --- | --- | --- | --- | --- | --- |
|  |  | **Univariate analysis** | |  | **Multivariate analysis** | |
| **Variables** |  | **HR (95% CI)** | ***P value*** |  | **HR (95% CI)** | ***P value*** |
| SMAD4 expression | No expression  at any site | 2.261 (0.786-6.502) | 0.130 |  | 1.240 (0.382-4.024) | 0.720 |
| Age (year) | >70 | 0.668 (0.343-1.301) | 0.235 |  | - |  |
| Gender | Men | 1.172 (0.561-2.448) | 0.673 |  | - |  |
| Location | Perihilar | 1.378 (0.704-2.700) | 0.349 |  | - |  |
| Histological type | Poorly | 1.438 (0.673-3.071) | 0.347 |  | - |  |
| Microinvasion into lymphatic system | Present | 1.853 (0.906-3.790) | 0.091 |  | 1.716 (0.813-3.619) | 0.156 |
| Microinvasion into venous system | Present | 2.034 (1.042-3.967) | **0.037** |  | 1.237 (0.607-2.519) | 0.558 |
| Microinvasion into nervous system | Present | 4.711 (1.435-15.462) | **0.011** |  | 4.250 (1.223-14.768) | **0.023** |
| Invasion into liver | Present | 1.828 (0.926-3.606) | 0.082 |  | 1.103 (0.532-2.287) | 0.791 |
| Invasion into pancreas | Present | 0.929 (0.466-1.850) | 0.834 |  | - |  |
| Invasion into portal vein | Present | 1.015 (0.358-2.878) | 0.977 |  | - |  |
| Invasion into artery | Present | 2.368 (0.976-5.743) | 0.057 |  | 1.039 (0.383-2.819) | 0.940 |
| UICC8th_pT | T3 or T4 | 1.342 (0.675-2.668) | 0.401 |  | - |  |
| UICC8th_pN | N1 or N2 | 2.152 (1.105-4.191) | **0.024** |  | 0.926 (0.425-2.020) | 0.848 |
| Residual tumor | R1 | 2.950 (1.490-5.841) | **0.002** |  | 2.860 (1.145-7.147) | **0.025** |
| Adjuvant therapy | Done | 0.932 (0.479-1.185) | 0.837 |  | - |  |
| Abbreviation; HR: hazard ratio CI: confidence interval  P value of <0.05 are indicated in bold. | | | | | | |

| **SUPPLEMENTAL TABLE 6**  Univariate and Multivariate analysis for overall survival of 73 patients with upfront-surgery group. | | | | | | |
| --- | --- | --- | --- | --- | --- | --- |
|  |  | **Univariate analysis** | |  | **Multivariate analysis** | |
| **Variables** |  | **HR (95% CI)** | ***P value*** |  | **HR (95% CI)** | ***P value*** |
| SMAD4 expression | No expression  at any site | 3.551 (1.204-10.473) | **0.022** |  | 2.487 (0.826-7.486) | 0.105 |
| Age (year) | >70 | 0.902 (0.427-1.908) | 0.788 |  |  |  |
| Gender | Men | 1.768 (0.714-4.376) | 0.218 |  |  |  |
| Location | Perihilar | 1.292 (0.610-2.733) | 0.504 |  |  |  |
| Histological type | Poorly | 0.787 (0.299-2.073) | 0.628 |  |  |  |
| Microinvasion into lymphatic system | Present | 3.136 (1.270-7.744) | **0.013** |  | 3.136 (1.160-8.475) | **0.024** |
| Microinvasion into venous system | Present | 1.745 (0.830-3.673) | 0.142 |  |  |  |
| Microinvasion into nervous system | Present | 5.269 (1.246-22.271) | **0.024** |  | 4.606 (1.050-20.198) | **0.043** |
| Invasion into liver | Present | 1.788 (0.845-3.780) | 0.128 |  |  |  |
| Invasion into pancreas | Present | 0.949 (0.444-2.030) | 0.893 |  |  |  |
| Invasion into portal vein | Present | 0.833 (0.251-2.761) | 0.765 |  |  |  |
| Invasion into artery | Present | 1.675 (0.580-4.834) | 0.340 |  |  |  |
| UICC8th_pT | T3 or T4 | 1.203 (0.563-2.571) | 0.633 |  |  |  |
| UICC8th_pN | N1 or N2 | 1.500 (0.715-3.148) | 0.284 |  |  |  |
| Residual tumor | R1 | 1.923 (0.886-4.174) | 0.098 |  | 2.254 (0.994-5.113) | 0.051 |
| Adjuvant therapy | Done | 0.915 (0.395-2.118) | 0.836 |  |  |  |
| Abbreviation; HR: hazard ratio CI: confidence interval  P value of <0.05 are indicated in bold. | | | | | | |

| **SUPPLEMENTAL TABLE 7**  Association between clinicopathological factors and neoadjuvant treatment. | | | | | | |  |
| --- | --- | --- | --- | --- | --- | --- | --- |
|  |  |  |  |  |  |  |  |
| **Variables** |  | **Upfront-surgery** |  | **NAC-RT^＊1^** |  | **NAC^＊2^** | ***P value*** |
| All |  | 73 |  | 21 |  | 4 |  |
| Gender | Men | 52 (71.2%) |  | 16 (76.2%) |  | 1 (25.0%) | 0.143 |
|  | Women | 21 (28.8%) |  | 5 (23.8%) |  | 3 (75.0%) |  |
| Age |  | 69.8±7.8^＊3^ |  | 63.0±9.3^＊3^ |  | 66.3±17.5^＊3^ | **0.007** |
| Operative procedure | Hepatectomy | 31 (46.3%) |  | 7 (33.3%) |  | 1 (25.0%) | 0.270 |
|  | Pancreaticoduodenectomy | 36 (53.7%) |  | 14 (66.7%) |  | 3 (16.7%) |  |
| Location | Perihilar | 31 (46.3%) |  | 7 (33.3%) |  | 1 (25.0%) | 0.270 |
|  | Distal | 36 (53.7%) |  | 14 (66.7%) |  | 3 (16.7%) |  |
| Histological type | Well or Moderately | 59 (80.8%) |  | 18 (85.7%) |  | 3 (75.0%) | 0.824 |
|  | Poorly | 14 (19.2%) |  | 3 (14.3%) |  | 1 (25.0%) |  |
| UICC8th_pT | 1 or 2 | 33 (45.2%) |  | 9 (42.9%) |  | 3 (75.0%) | 0.474 |
|  | 3 or 4 | 40 (54.8%) |  | 12 (57.1%) |  | 1 (25.0%) |  |
| UICC8th_pN | 0 | 42 (57.5%) |  | 18 (85.7%) |  | 1 (25.0%) | **0.013** |
|  | 1 or 2 | 31 (42.5%) |  | 3 (14.3%) |  | 3 (75.0%) |  |
| Microinvasion into lymphatic system | Absent | 30 (41.1%) |  | 16 (76.2%) |  | 1 (25.0%) | **0.010** |
|  | Present | 43 (58.9%) |  | 5 (23.8%) |  | 3 (75.0%) |  |
| Microinvasion into venous system | Absent | 48 (65.8%) |  | 18 (85.7%) |  | 3 (75.0%) | 0.175 |
|  | Present | 25 (34.3%) |  | 3 (14.3%) |  | 1 (25.0%) |  |
| Microinvasion into nervous system | Absent | 16 (21.9%) |  | 10 (47.6%) |  | 1 (25.0%) | 0.081 |
|  | Present | 57 (78.1%) |  | 11 (52.4%) |  | 3 (75.0%) |  |
| Invasion into liver | Absent | 51 (69.9%) |  | 17 (81.0%) |  | 4 (100.0%) | 0.166 |
|  | Present | 22 (30.1%) |  | 4 (19.1%) |  | 0 (0.0%) |  |
| Invasion into pancreas | Absent | 46 (63.0%) |  | 12 (57.1%) |  | 3 (75.0%) | 0.763 |
|  | Present | 27 (37.0%) |  | 9 (42.9%) |  | 1 (25.0%) |  |
| Invasion into portal vein | Absent | 66 (90.4%) |  | 17 (91.0%) |  | 4 (100.0%) | 0.324 |
|  | Present | 7 (9.6%) |  | 4 (9.0%) |  | 0 (0.0%) |  |
| Invasion into artery | Absent | 66 (91.7%) |  | 19 (90.5%) |  | 4 (100.0%) | 0.693 |
|  | Present | 6 (8.3%) |  | 2 (9.5%) |  | 0 (0.0%) |  |
| Residual tumor | R0 | 66 (90.4%) |  | 21 (100.0%) |  | 4 (100.0%) | 0.116 |
|  | R1 | 7 (9.6%) |  | 0 (0.0%) |  | 0 (0.0%) |  |
| Adjuvant therapy | Done | 32 (43.8%) |  | 13 (61.9%) |  | 1 (25.0%) | 0.224 |
|  | Not done | 41 (56.2%) |  | 8 (38.1%) |  | 3 (75.0%) |  |
| ＊1: cases treated with neoadjuvant chemo-radiotherapy ＊2: cases treated with neoadjuvant chemotherapy  ＊3: average ± standard deviation  P value of <0.05 are indicated in bold | | | | | | | |
